# Which Socio-Ecological Factors Associate with a Switch to or Maintenance of Active and Passive Transport during the Transition from Primary to Secondary School?

**DOI:** 10.1371/journal.pone.0156531

**Published:** 2016-05-27

**Authors:** Griet Vanwolleghem, Delfien Van Dyck, Femke De Meester, Ilse De Bourdeaudhuij, Greet Cardon, Freja Gheysen

**Affiliations:** 1 Department of Movement and Sport Sciences, Faculty of Medicine and Health Sciences, Ghent University, Ghent, Belgium; 2 Research Foundation Flanders (FWO), Brussels, Belgium; 3 Vlaams Instituut voor Gezondheidspromotie en Ziektepreventie (VIGeZ), Brussels, Belgium; University of New South Wales, AUSTRALIA

## Abstract

**Objectives:**

The aim was to investigate which individual, psychosocial and physical neighborhood environmental factors associate with children’s switch to or maintenance of active/passive transport to school and to leisure time destinations during the transition from primary to secondary school.

**Methods:**

Children (n = 313) filled out a questionnaire in the last year of primary school and 2 years later to assess socio-demographic characteristics and self-reported transport. One of their parents completed a questionnaire to assess parental perceptions of psychosocial and physical neighborhood environmental factors.

**Results:**

The increase of the home-school distance was significantly associated with children’s switch to or maintenance of passive transport to school compared to a switch to (OR = 0.81; p = 0.03) and maintenance (OR = 0.87; p = 0.03) of active transport to school. Low SES was associated with children’s switch to active transport to school compared to maintenance of active transport (OR = 3.67; p = 0.07). For transport to leisure time destinations, other factors such as parental perceived neighborhood safety from traffic and crime (OR = 2.78; p = 0.004), a positive social norm (OR = 1.49; p = 0.08), positive attitudes (OR = 1.39; p = 0.08) (i.e. more benefits, less barriers) towards their children’s physical activity and poor walking/cycling facilities in the neighborhood (OR = 0.70; p = 0.06) were associated with children’s maintenance of active transport to leisure time destinations compared to a switch to or maintenance of passive transport.

**Conclusions:**

This longitudinal study can give directions for interventions promoting children’s active transport during the transition to secondary school. It is necessary to promote different possibilities at primary school for children to use active transport when going to secondary school. Walking/cycling a part of the home-school trip can be a possible solution for children who will be living at non-feasible distances from secondary school. Providing safe neighborhoods, combined with programs for parents stimulating a positive social norm and positive attitudes towards physical activity during primary school, can be effective.

## Introduction

Engaging in walking and cycling provides numerous health-benefits [1] and encourages the development of different social and motor skills [2] among children and adolescents. Moreover, active transport to school and to leisure time destinations have been identified as important targets for increasing physical activity levels in children and adolescents [3–5].

The transition from primary (age 11–12 years) to secondary school (age 13–14 years) is an important time period for children, characterized by major changes such as changing school and becoming more independent. In this time period, (un)healthy behaviors can be developed or sustained and these behaviors may further track into adulthood [6]. Children may maintain using active transport when they grow older, switch to active transport, switch to or maintain using passive transport (being dropped off by car, using public transport) [7]. Overall, active transport in European and Australian children has been found to increase moderately (+5.5 min/day of active transport to school [8]; +1.5 trips/week of active transport in leisure time [9]) during the transition from primary to secondary school [7–10] due to increases in independent mobility [8,11]. However, during the transition from primary to secondary school, many children, often living within feasible distances for active transport, switch to or keep using passive transport to go to school or leisure time destinations [7,10]. In order to prevent a switch to or maintenance of passive transport during the transition from primary to secondary school, it is crucial to understand the contributing factors.

To examine these correlates, a socio-ecological perspective can be used. Socio-ecological models emphasize that besides individual factors, (psycho)social and environmental factors can be important for children’s and adolescents’ physical activity [12]. Furthermore, research focusing on only one type of factors (e.g. only physical environmental), without including other factors (e.g. psychosocial), may underestimate the strength of the influences affecting children’s and adolescents’ active transport [12]. In a recent review, D’Haese and colleagues (2015) [13] concluded that there is a specific need for studies focusing on the combination of individual, psychosocial and environmental factors of children’s active transport behavior [13]. Moreover, the studies that have been conducted until now were cross-sectional which makes it difficult to draw causal conclusions [14–19]. To our knowledge, only two longitudinal studies, conducted in the UK and Australia, investigated individual, psychosocial and physical environmental factors of children’s switch to or maintenance of active and passive transport during the transition from primary to secondary school [7,10]. Both studies focused only on transport to school and did not investigate the factors of transport to leisure time destinations. Factors influencing children’s switch or maintenance of transport modes to leisure time destinations may be different than those influencing transport to school [20], since transport to leisure time destinations is less mandatory and less prone to time constraints [21]. Additionally, only Panter and colleagues (2013) included measures of passive transport. Clear knowledge of the factors that motivate children to switch to or maintain using passive transport can be relevant when developing interventions promoting active transport.

In order to meet the above limitations in the current literature, the aim of the present study was to investigate which individual, psychosocial and physical neighborhood environmental factors associate with children’s switch to or maintenance of active/passive transport to school and to leisure time destinations during the transition from primary (11–12 years) to secondary school (13–14 years). Different types of behavior transition were determined: (1) switching to active transport, (2) maintaining active transport, (3) switching to or maintaining passive transport.

## Materials and Methods

### Participants and Procedure

During the school year 2009–2010, a convenience sample of 148 primary schools in two regions (East- and West-Flanders) of Flanders (northern part of Belgium) was contacted by phone and 44 primary schools agreed to participate (response rate = 29.7%). In each participating school, one class from the 6^th^ grade (11–12 year old) was randomly selected and children (n = 976) were invited to participate in the study. Written parental informed consent was obtained from the parents of 749 children (76.7%). With this consent, parents gave permission for themselves and for their child’s participation in the study. After a written consent was received, a researcher went to the different classes with participating children. During the class visits, children were asked to complete a questionnaire (see S1 File) including socio-demographic information and active transport behavior under the supervision of a researcher. Since parents still play an important role to let their child walk or cycle independently despite children’s increase of independent mobility [22], one of the parents of the children was asked to complete a questionnaire (see S2 File) at home including socio-demographic information, parental perceived psychosocial factors towards their child’s physical activity and parental perceptions of the neighborhood environment. In total, 736 children (98.3%) and 701 parents (93.5%) completed the questionnaires.

Two years later, parents of the children (n = 736) who participated at baseline (primary school) were contacted by phone and 502 agreed to participate in the follow-up study (response rate = 68.2%). Written parental informed consent to give permission for themselves and for their child to participate in the follow-up measurements of the study was first obtained. After a written consent was received, children and parents received a similar child and parental questionnaire as during baseline measurements via regular mail. Parents and children were asked to send the questionnaires back. In total, 420 children (83.7%) and 416 parents (82.8%) completed the questionnaires. Complete data at baseline and follow-up from both children and parents were obtained from 321 children and parent pairs. Children who moved residence between baseline and follow-up were excluded from the dataset in order to exclude the potential influences of different neighborhood and home environments between baseline and follow-up (n = 8). In total, 313 children (and parents) were included in the analyses. Drop out analyses (t-test and X^2^ tests) showed no significant differences in baseline characteristics (age (t = -0.35; p = 0.73), sex (X^2^ = 0.11; p = 0.74), socio-economic status (SES) (X^2^ = 0.09; p = 0.76), active/passive transport to school (X^2^ = 1.71; p = 0.19), active/passive transport to leisure time destinations (X^2^ = 0.01; p = 0.95)) between the sample of children included in the present study (n = 313) and the sample of children who dropped out (n = 423) (drop-out from baseline to follow-up, no complete data from children and parent pairs, moved residence between baseline and follow-up).

The present study was approved by the Ethics Committee of the Ghent University Hospital (EC UZG 2011/208 B670201112641).

### Measurements

#### Socio-demographic information

The child questionnaire contained general questions about the child (age, sex). The educational level of the parents was used as a proxy measure of children’s SES. The educational level was based on four options: did not complete secondary school, completed secondary school, completed college, or completed university. Children were identified as being of high SES when at least one parent reached a college or university level.

Home-school distances at baseline and follow-up (in km) were assessed by a researcher, defining the shortest route between the home and (primary or secondary) school address on a street map using the street network. Home addresses were received through the parental questionnaires and school addresses through the corresponding school principals. Since children changed schools during the transition from primary to secondary school, the difference in home-school distance (in km) between primary and secondary school was calculated by the following formula: shortest distance from home to secondary school (km)–shortest distance from home to primary school (km).

Parental perceived psychosocial factors. The parental questionnaire contained questions to assess parental perceived psychosocial factors towards children’s physical activity behavior. Based on the ASE-model [23], the following four subscales were calculated: parental support, social norm, self efficacy of their child’s physical activity and attitude towards their child’s physical activity. The questions that were used to assess the different subscales were based on previous studies in children and adolescents [24–25] (see Table 1 for an outline of the questionnaire). Each subscale contained multiple questions, except for the subscales ‘parental support’ and ‘social norm’ which were measured by one item. Response options for the question to obtain the subscale parental support were scored on a 5-point scale, ranging from never to very often. Response options for the questions to obtain the other subscales were also 5-point scales, ranging from totally unimportant/disagree to totally important/agree. Subscales with multiple questions were scored by taking the mean of the different question scores. To calculate the subscale ‘attitude’, the mean of questions containing barriers towards their child’s physical activity was subtracted from the mean of questions containing benefits towards their child’s physical activity. Internal consistency for the subscales containing multiple questions was found to be acceptable (respectively 0.54 for attitude, 0.84 for self efficacy).

**Table 1:** Content and response options of the parental perceived psychosocial and physical neighborhood environmental factors.

	Content of the items	Response options
**Psychosocial factors**^1^		
Parental support (1 item)	How frequently do you encourage your child to be physically active?	never, seldom, sometimes, often, very often
Social norm (1 item)	My child has to participate regularly in physical activity.	strongly disagree, somewhat disagree, neither agree or disagree, somewhat agree, strongly agree
Self efficacy (4 items: Cronbach Alpha = 0.84)	I am sure my child will be physically active if…	strongly disagree, somewhat disagree, neither agree or disagree, somewhat agree, strongly agree
	a. he/she has to get up early.	
	b. his/her friends want to do something else.	
	c. he/she has a lot of work for school.	
	d. it is exhausting and difficult.	
Attitude = Benefits-barriers (11 items: Cronbach Alpha = 0.54)	Benefits: My child thinks that doing sports is good because…	strongly disagree, somewhat disagree, neither agree or disagree, somewhat agree, strongly agree
	a. he/she improves his/her condition and health.	
	b. he/she gets in contact with (new) friends.	
	c. he/she enjoys being physically active.	
	d. he/she can show that he/she is better in sports than others.	
	e. he/she does not get bored if he/she is physically active.	
	f. he/she loses weight.	
	Barriers: My child is not able to engage in sports because…	strongly disagree, somewhat disagree, neither agree or disagree, somewhat agree, strongly agree
	g. of lack of time.	
	h. he/she does not enjoy sports.	
	i. he/she is not good in doing sports.	
	j. my child does not have transportation to engage in sports.	
	k. he/she is not allowed to sport by his/her parents.	
**Physical neighborhood environmental factors**^2^		
Residential density (3 items)	a. How common are separate or standalone one family homes in your neighborhood?^a^	None, a few, about half, a lot, all
	b. How common are connected townhouses or row houses in your neighborhood?^a^	
	c. How common are apartments in your neighborhood?^a^	
Land use mix diversity (8 items)	How long does it take (for your child) to walk from your home to…	> 30 min, 21–30 min, 11–20 min, 6–10 min, 1–5 min
	a. Grocery store^a^	
	b. Supermarket^a^	
	c. Bakery	
	d. Butchery	
	e. Convenience store^a^	
	f. Bank^a^	
	g. Library^a^	
	h. My school/school of my child^a^	
Street connectivity (2 items)	a. The streets in our neighborhood have many cul-de-sacs (dead end streets).^a^	strongly disagree, somewhat disagree, neither agree or disagree, somewhat agree, strongly agree
	b. There are a lot of crossroads in my neighborhood.^a^	
Land use mix access (4 items)	a. In my neighborhood it’s easy (for my child) to walk to school.	strongly disagree, somewhat disagree, neither agree or disagree, somewhat agree, strongly agree
	b. There are many places (for my child) to go (alone or with someone) within easy walking distance of my home.^a^	
	c. In my neighborhood it’s easy (for my child) to get from place to place (for example, freeways, railway lines, rivers).^a^	
	d. In my neighborhood it’s easy (for my child) to walk to a playground, park or skate park from my house.	
Walking/cycling facilities (9 items)	a. There are sidewalks on most of the streets in my neighborhood.^a^	strongly disagree, somewhat disagree, neither agree or disagree, somewhat agree, strongly agree
	b. There are cycle lanes on most of the streets in my neighborhood.	
	c. Cycle lanes are separated from the road/traffic in my neighborhood by parked cars or grass.	
	d. There are bicycle racks in my neighborhood (at shops, schools, transit stops, …).	
	e. At night the sidewalks are well-lit in my neighborhood.	
	f. The sidewalks are well maintained in my neighborhood.	
	g. At night the cycle lanes are well-lit in my neighborhood.	
	h. The cycle lanes are well maintained in my neighborhood.	
	i. Playground and parks are well maintained in my neighborhood.	
Aesthetics (3 items)	a. There are trees along the streets in my neighborhood.^a^	strongly disagree, somewhat disagree, neither agree or disagree, somewhat agree, strongly agree
	b. There are many beautiful natural things (for my child) to look at in my neighborhood (e.g. gardens, views).^a^	
	c. There are many buildings/homes in our neighborhood that are nice (for my child) to look at.^a^	
Safety (10 items)	a. There is so much traffic along nearby streets that it makes it difficult or unpleasant (for my child) to walk (alone or with someone) in my neighborhood.^a^	strongly disagree, somewhat disagree, neither agree or disagree, somewhat agree, strongly agree
	b. There is so much traffic along nearby streets that it makes it difficult or unpleasant for my child to cycle (alone or with someone) in my neighborhood.	
	c. The speed of traffic on most nearby streets is usually slow.^a^	
	d. Our neighborhood streets have good lighting at night.^a^	
	e. There are crosswalks and signals to help walkers cross busy streets in our neighborhood.^a^	
	f. It’s safe for my child to play on the street in my neighborhood.	
	g. There is a low crime rate in our neighborhood.^a^	
	h. I am worried about (letting my child) play(ing) outside alone around my home (e.g. yard, driveway, apartment common area) because I am afraid of them being taken or hurt by a stranger.^a^	
	i. I am worried about (letting my child) be(ing) alone in a local or nearby park because I am afraid of them being taken or hurt by a stranger.^a^	
	j. My bike is securely locked in my neighborhood.	
Recreational facilities (5 items)	How long does it take (for your child) to cycle from your home to…	> 30 min, 21–30 min, 11–20 min, 6–10 min, 1–5 min
	a. Indoor recreation facility^a^	
	b. Outdoor recreation facility^a^	
	c. Public park^a^	
	d. Swimming pool^a^	
	e. Public playground^a^	

^1^parental perceived psychosocial factors towards their child’s physical activity at baseline (primary school);

^2^parental perceived physical neighborhood environmental factors at baseline (primary school)

^a^questions derived from the parent version of NEWS-Y (Rosenberg et al., 2009 [26])

#### Parental perceived physical neighborhood environmental factors

Parental perceptions of the neighborhood environment were based on the parent version of the Neighborhood Environmental Walkability Scale for Youth (NEWS-Y) [26] with the addition of some questions to comply with the Belgian environment (see Table 1 for an outline of the questionnaire). The following subscales were calculated: residential density, land use mix diversity, land use mix access, street network connectivity, availability and quality of walking and cycling facilities, aesthetics, perceived safety from traffic and crime, convenience of recreational facilities in the neighborhood. Each subscale contained multiple questions. Response options for the three questions to obtain the subscale neighborhood residential density were scored on a 5-point scale, ranging from none to all. Standardized scoring guidelines of the NEWS-Y were used to calculate the different subscales [27]. Since connected townhouses, row houses and apartments are considered to be more person-dense than separate or standalone one family homes, the residential density items were weighted relative to the average density of separate or standalone one family homes [28]. The subscale neighborhood residential density was then calculated by the following formula: score on question 1a (separate or standalone one family homes) + 12*score on question 1b (connected townhouses or row houses) + 25*score on question 1c (apartments) [27]. Response options to obtain the subscales land use mix diversity and convenience of recreational facilities in the neighborhood were: > 30 min, 21–30 min, 11–20 min, 6–10 min, 1–5 min. Response options for the questions regarding the other subscales were scored on a 4-point scale, ranging from strongly disagree to strongly agree. The remaining subscales (land use mix diversity, land use mix access, street network connectivity, availability and quality of walking and cycling facilities, aesthetics, safety from traffic and crime, convenience of recreational facilities in the neighborhood) were scored by taking the mean of the different question scores [27]. Land use mix diversity, street connectivity and residential density are often combined into a walkability index [29]. Perceived neighborhood walkability was obtained by using an adapted version of the standardized formula of Frank and colleagues [30]: walkability z-score = z-score residential density + 2*z-score connectivity + z-score land use mix [30]. Based on evaluated weighting schemes, the street connectivity z-score was weighted by a factor of two within the walkability index due to the strong influence of street connectivity on non-motorized transport [29]. Internal consistency for the six subscales used in the analyses of the present paper (perceived walkability, land use mix access, availability and quality of walking and cycling facilities, aesthetics, safety, convenience of recreational facilities in the neighborhood) was found to be acceptable (ranging from 0.54 to 0.89).

#### Self-reported transport to school and to leisure time destinations

Self-reported transport at baseline and at follow-up was used to identify different types of children’s active or passive transport to school and to leisure time destinations during the transition from primary to secondary school. The Flemish Physical Activity Questionnaire (FPAQ) [31] was used to assess children’s self-reported transport to school and to leisure time destinations. The FPAQ was found to be a reliable and valid questionnaire to assess different domains of physical activity in children [31–32].

Specifically, children (together with their parents) were asked at baseline and at follow-up: “How do you usually go to school?”. Response options were: on foot or by bike (active transport to school), dropped off by car or using the public transport (passive transport to school). To assess children’s transport to leisure time destinations, children were asked if they walked or cycled for transport (excluding transport to school or recreational walking/cycling) during the last week (including weekend). Response options were: yes (active transport to leisure time destinations) or no (passive transport to leisure time destinations).

### Data Analysis

The Statistical Package for the Social Sciences for Windows version 21 (SPSS Inc., Chicago, IL, USA) was used to describe and analyze the characteristics of the sample. Means, standard deviations (SD) and percentages were used to describe the sample and transport behavior.

To identify different types of active and passive transport to school and to leisure time destinations during the transition from primary to secondary school, the different transport modes at baseline and at follow-up were used. Children were classified into one of three types of behavior transition for transport to school: (1) switching to active transport, (2) maintaining active transport and (3) switching to or maintaining passive transport. For transport to leisure time destinations, only two types ((1) maintaining active transport, (2) switching to or maintaining passive transport) were further used in the analyses due to insufficient numbers in the type ‘switch to active transport’ (n = 14). The types of behavior transition ‘switching to or maintaining passive transport’ were taken together in the present study since both represent the less favorable behavior. The three types of behavior transition for transport to school and two types of behavior transition for transport to leisure time destinations were used as main outcomes in the analyses.

To investigate the individual, psychosocial and physical neighborhood environmental factors of children’s switch to or maintenance of active/passive transport to school and to leisure time destinations, 2-level (school-child) multinomial regression models were constructed in SPSS allowing for clustering at the baseline school level. Individual (sex, SES), parental perceived psychosocial factors (parental support, social norm, self efficacy, attitude) and parental perceived physical neighborhood environmental factors (perceived walkability, land use mix access, walking and cycling facilities, aesthetics, safety, recreational facilities in the neighborhood) were used as independent variables in the analyses. Variables on a 5-point Likert-scale were treated as continuous [33–34] and were centered by grand mean in the multinomial logistic regression analyses. Independent variables were checked for multicollinearity and no high correlations (r>0.60) between the variables were found. Since the distance between home and school is an important correlate of active transport to school among children and because children changed schools during the transition from primary to secondary school, the difference in home-school distance (in km) between primary and secondary school was also included as an independent variable in the analyses for transport to school. Analyses were controlled for children’s baseline transport behavior (primary school), separately for transport to school and transport to leisure time destinations. The significance level was set at p <0.05. A trend to significance was defined for 0.05≤p<0.10.

## Results

### Description of Sample and Transport Behavior

Of the 313 children, 51.1% (n = 160) were boys. In total, 63.3% (n = 197) had a high SES. Mean age was 11.0 ± 0.5 years at baseline, and 13.4 ± 0.6 years at follow-up. At baseline and follow-up, respectively 65.2% (n = 204) and 65.5% (n = 205) used active transport to school. Cycling was the most common transport mode (44.4% (n = 139) at baseline, 58.5% (n = 183) at follow-up). Furthermore, 93.9% (n = 294) used active transport to leisure time destinations at baseline and 70.0% (n = 219) at follow-up. For transition of transport to leisure time destinations, 65.5% (n = 205) of the children maintained using active transport, 4.5% (n = 14) switched from passive to active transport and 30% (n = 94) switched to or maintained using passive transport.

Children who switched from passive to active transport to school (n = 58; 18.5%) lived on average 3.8 ± 3.1 km from primary school and 3.9 ± 3.2 km from secondary school. Children who maintained using active transport to school (n = 146; 46.7%) lived on average 1.5 ± 1.7 km from primary school and 3.7 ± 4.8 km from secondary school. Children who switched to or maintained using passive transport to school (n = 109; 34.8%) lived on average 3.0 ± 4.5 km from primary school and 8.6 ± 6.3 km from secondary school.

### Individual, Psychosocial and Physical Neighborhood Environmental Factors of Switch to/Maintenance of Transport to School during the Transition to Secondary School

Results of the multinomial logistic regression model for transport to school are shown in Table 2. Difference in distance between primary and secondary school was found to be significantly associated with children’s switch to or maintenance of active/passive transport to school. The higher the increase in distance between primary and secondary school, the less likely children switched to (OR = 0.81; p = 0.03) or maintained using active transport to school (OR = 0.87; p = 0.03) compared to switching to or maintaining passive transport to school. SES was found to be marginally significantly associated: children of low SES were more likely (OR = 3.67; p = 0.07) to switch to active transport to school compared to maintain using active transport to school. None of the other factors were significantly associated with children’s switch to or maintenance of active/passive transport to school.

**Table 2:** Results of the 2-level multinomial regression model for transport to school.

	Switching to active transport to school (n = 58)	Maintaining active transport to school (n = 146)	Switching to or maintaining passive transport to school (n = 109)	Switching to active transport to school—Switching to or maintaining passive transport to school°			Maintaining active transport to school—Switching to or maintaining passive transport to school°			Switching to active transport to school—Maintaining active transport to school°		
***Individual factors***												
	n (%)	n (%)	n (%)	OR	p	95% CI	OR	p	95% CI	OR	p	95% CI
Sex												
*Boys*	24 (41.4)	85 (58.2)	51 (46.8)	1.13	0.81	0.41–3.16	1.43	0.40	0.62–3.29	0.78	0.72	0.21–2.96
*Girls*°	34 (58.6)	61 (41.8)	58 (53.2)									
SES												
*Low*	18 (31.0)	52 (35.9)	44 (40.7)	2.00	0.24	0.63–6.32	0.59	0.20	0.26–1.32	3.67	0.07	0.98–7.28
*High*°	40 (69.0)	93 (64.1)	64 (59.3)									
***Psychosocial factors***^1^												
	Mean (SE)	Mean (SE)	Mean (SE)									
Parental support	3.98 (0.12)	3.87 (0.08)	3.70 (0.09)	1.11	0.83	0.42–2.92	1.33	0.16	0.90–1.96	0.88	0.85	0.25–3.10
Social norm	4.71 (0.07)	4.71 (0.05)	4.75 (0.05)	1.47	0.60	0.35–6.14	0.97	0.94	0.45–2.10	1.46	0.67	0.26–8.10
Self efficacy	3.68 (0.12)	3.68 (0.07)	3.64 (0.08)	0.67	0.28	0.32–1.40	1.33	0.43	0.65–2.72	0.52	0.16	0.21–1.29
Attitude	1.80 (0.13)	1.81 (0.08)	1.73 (0.09)	1.63	0.13	0.87–3.07	0.79	0.58	0.35–1.81	2.01	0.17	0.75–5.40
***Physical neighborhood environmental factors***^2^												
	Mean (SE)	Mean (SE)	Mean (SE)									
Walkability z-score	-0.47 (0.37)	0.48 (0.23)	-0.48 (0.29)	1.12	0.43	0.85–1.47	1.00	0.86	0.84–1.15	1.15	0.35	0.86–1.54
Land use mix access	2.93 (0.12)	3.86 (0.09)	3.42 (0.11)	1.10	0.62	0.75–1.62	0.78	0.38	0.46–1.35	1.40	0.26	0.78–2.53
Walking/cycling facilities	2.88 (0.10)	3.10 (0.07)	2.81 (0.09)	0.97	0.92	0.53–1.77	1.36	0.46	0.59–3.14	0.67	0.37	0.28–1.61
Aesthetics	3.28 (0.11)	3.18 (0.07)	3.37 (0.07)	1.19	0.66	0.54–2.65	0.83	0.41	0.54–1.28	1.55	0.37	0.60–4.03
Safety	3.08 (0.07)	3.15 (0.05)	3.06 (0.05)	1.24	0.69	0.44–3.51	1.04	0.92	0.54–1.98	1.51	0.54	0.40–5.69
Recreational facilities	3.39 (0.12)	3.61 (0.07)	3.26 (0.09)	1.16	0.69	0.56–2.44	1.37	0.18	0.87–2.16	0.92	0.86	0.37–2.28
Difference home-school distance (km)^#^	0.14 (0.40)	2.22 (0.37)	5.73 (0.67)	**0.81**	**0.03**	**0.67–0.97**	**0.87**	**0.03**	**0.77–0.98**	0.95	0.60	0.77–1.16

°Reference category

OR = Odds ratio; CI = Confidence Interval; SES = Social economic status; SE = Standard Error

^1^parental perceived psychosocial factors towards their child’s physical activity at baseline (primary school);

^2^parental perceived physical neighborhood environmental factors at baseline (primary school)

^#^ shortest distance from home to secondary school (km)–shortest distance from home to primary school (km)

Analyses controlled for baseline-level active transport to school (at primary school)

**Bold = significant (p<0.05)**; Underlined = trend to significance (0.05≤p<0.10)

### Individual, Psychosocial and Physical Neighborhood Environmental Factors of Switch to/Maintenance of Transport to Leisure Time Destinations during the Transition to Secondary School

Results of the multinomial logistic regression model for transport to leisure time destinations are shown in Table 3. None of the individual factors were significantly associated with children’s switch to or maintenance of active/passive transport to leisure time destinations.

**Table 3:** Results of the 2-level multinomial regression model for transport to leisure time destinations.

	Maintaining active transport to leisure time destinations (n = 205)	Switching to and maintaining passive transport to leisure time destinations (n = 94)°			
***Individual factors***					
	n (%)	n (%)	OR	p	95% CI
Sex			0.77	0.30	0.48–1.26
*Boys*	101 (49.3)	51 (54.3)			
*Girls*°	104 (50.7)	43 (45.7)			
SES			0.57	0.10	0.29–1.11
*Low*	67 (32.7)	42 (45.2)			
*High*°	138 (67.3)	51 (54.8)			
***Psychosocial factors***^1^					
	Mean (SE)	Mean (SE)			
Parental support	3.86 (0.06)	3.87 (0.10)	0.95	0.78	0.67–1.35
Social norm	4.75 (0.04)	4.66 (0.07)	1.49	0.08	0.98–2.46
Self efficacy	3.63 (0.06)	3.68 (0.09)	0.71	0.24	0.41–1.26
Attitude	1.81 (0.07)	1.66 (0.10)	1.39	0.08	0.97–2.00
***Physical neighborhood environmental factors***^2^					
	Mean (SE)	Mean (SE)			
Walkability z-score	-0.25 (0.20)	0.71 (0.32)	0.97	0.59	0.88–1.08
Land use mix access	3.44 (0.08)	3.79 (0.11)	0.76	0.16	0.52–1.12
Walking/cycling facilities	2.93 (0.06)	3.06 (0.08)	0.70	0.06	0.48–1.01
Aesthetics	3.30 (0.06)	3.13 (0.08)	0.96	0.81	0.68–1.35
Safety	3.17 (0.04)	2.99 (0.06)	**2.78**	**0.004**	**1.39–5.53**
Recreational facilities	3.37 (0.06)	3.66 (0.07)	0.78	0.22	0.54–1.16

°Reference category

OR = Odds ratio; CI = Confidence Interval; SES = Social economic status; SE = Standard Error

^1^parental perceived psychosocial factors towards their child’s physical activity at baseline (primary school);

^2^parental perceived physical neighborhood environmental factors at baseline (primary school)

Analyses controlled for baseline-level active transport to leisure time destinations (at primary school)

**Bold = significant (p<0.05)**; Underlined = trend to significance (0.05≤p<0.10)

For parental perceived social norm and attitude towards physical activity at baseline, a trend towards significance was found. The more parents perceived the importance for their child to participate regularly in physical activity (social norm), the more likely children maintained using active transport to leisure time destinations (OR = 1.49; p = 0.08) compared to switching to or maintaining passive transport to leisure time destinations. Furthermore, the higher the perceived attitudes from parents (more benefits, less barriers) towards physical activity at baseline, the more likely children maintained using active transport to leisure time destinations (OR = 1.39; p = 0.08) compared to switching to or maintaining passive transport to leisure time destinations. No significant results were found for the other parental perceived psychosocial factors at baseline.

Parental perceived neighborhood safety at baseline was found to be significantly associated with children’s switch to or maintenance of active/passive transport in leisure time. The higher the parental perceived neighborhood safety at baseline, the more likely children maintained using active transport to leisure time destinations (OR = 2.78; p = 0.004) compared to switching to or maintaining passive transport to leisure time destinations. Parental perceived walking and cycling facilities in the neighborhood at baseline was found to be marginally significantly associated: the higher the parental perceived walking and cycling facilities in the neighborhood at baseline, the less likely children maintained using active transport to leisure time destinations (OR = 0.70; p = 0.06) compared to switching to or maintaining passive transport to leisure time destinations. No significant results were found for the other parental perceived physical neighborhood environmental factors at baseline.

## Discussion

When examining children’s transport behavior during the transition from primary to secondary school, the highest prevalence was found for “maintaining active transport” to school (46.7%) and to leisure time destinations (65.5%) compared to the other behavior transitions. These results are in line with previous research that demonstrated that active transport tracks into adolescence [35]. Nevertheless, in our study still a lot of children switched to or maintained using passive transport to school (34.8%) and to leisure time destinations (30.0%). The findings of the present study are crucial to understand why children switch to or keep using active or passive transport. In general, the results indicated that a few individual, parental perceived psychosocial and physical neighborhood environmental factors were associated with children’s switch to or maintenance of active/passive transport to school and to leisure time destinations. More specifically, for transport to leisure time destinations, more and different associations were identified than for transport to school.

We found that the increase of the home-school distance was the only significant factor associated with children’s switch to or maintenance of passive transport to school, which is in line with two other longitudinal studies investigating the correlates of children’s transport behavior to school during the transition from primary to secondary school [7,10]. The result of our longitudinal study confirmed that home-school distance is a key factor for children’s change in active transport behavior. Regardless of other individual, psychosocial and physical neighborhood environmental factors, children were more likely to switch to or maintain using passive transport to school when the home-school distance between primary and secondary school increased more strongly (average increase of 5.7 km). The average increase of the home-school distance between primary and secondary school was less pronounced for children who switched to (+0.14 km) or kept using (+2.22 km) active transport to school. From a public health perspective it seems important to limit home-school distances (e.g. merging schools with a large area to serve should not be advised), but this is not always feasible. However, also children living at more feasible active transport distances (<3 km [36]) from secondary school switched to or maintained using passive transport to school in our study. To know why these children switched to or kept using passive transport to school, a comparison of correlates between children living at feasible and non-feasible active transport distances from school are of interest. However, this implies further subgroup analyses (based on different home-school distances) with larger sample sizes than in the current study.

Besides distance to school, only one baseline factor determined children’s switch to active transport to school during the transition to secondary school: children of low SES were more likely to switch from passive to active transport to school than to maintain using active transport to school. Panter and colleagues (2013) also found that children of low SES were more likely to take up walking and cycling [7]. It is known that financial problems are a barrier for passive transport (car or public transport) for low SES children [37]. However, in primary school, it seems that other barriers (e.g. safety concerns) outweigh the financial barriers in low SES parents, which may explain why some parents still drive their child to school. In secondary school, it is likely that those children are more able to deal with unsafe situations and therefore use active transport to go to school, which could explain the switch from passive to active transport in low SES children.

Parental perceptions of parental support, attitudes, social norm and self efficacy towards their child’s physical activity at primary school were not significantly associated with children’s switch to or maintenance of active/passive transport to school. However, in our study the questions to assess parental perceived psychosocial factors were developed towards children’s physical activity in general (e.g. How frequently do you encourage your child to be physically active?). Possibly, if the questions would have been specifically directed towards children’s transport behavior to school, these psychosocial factors could have emerged as potential correlates.

For transport to leisure time destinations, parental perception of neighborhood safety from traffic and crime at baseline was a significant factor for children to maintain using active transport to leisure time destinations compared to switching to or maintaining passive transport to leisure time destinations. Until now, longitudinal studies on the associations with children’s switch to and maintenance of active/passive transport to leisure time destinations are scarce [9]. The only longitudinal study [9] examining associations with children’s transport to leisure time destinations during the transition to secondary school did not include safety factors. Additionally, Carver and colleagues included only a few physical neighborhood environmental factors (i.e. only factors evaluating the road environment) and not in combination with other individual and psychosocial factors.

In addition to neighborhood safety, a surprising and contrasting significant result was found for parental perceived walking and cycling facilities in the neighborhood. Children were more likely to switch to or maintain using passive transport to leisure time destinations when their parents perceived better walking and cycling facilities in the neighborhood at baseline. Previous cross-sectional studies reported inconclusive results regarding the contribution of parental perceived walking and cycling facilities to children’s transport behavior [13,26]. A possible explanation for our finding could lie in the fact that the neighborhoods with good walking and cycling facilities were perceived as less safe. To illustrate, major roads possibly have better walking and cycling facilities (e.g. separated cycle lanes) than more rural roads where walking and cycling facilities are often lacking. It may be the case that adolescents mainly make use of such major roads (that are probably less safe due to higher traffic volume) to travel larger distances to leisure time destinations. This could explain why children switch to or keep using passive transport to leisure time destinations. Moreover, it is possible that other (environmental) factors besides walking and cycling facilities in the neighborhood are also important to explain children’s maintenance of active transport to leisure time destinations (e.g. road environment).

In our study, we also found that parental perceived social norm and attitudes towards their children’s physical activity at primary school were positively associated with maintaining active transport to leisure time destinations compared to switching to or maintaining passive transport. In general, the current literature lacks information on the psychosocial correlates of transport to leisure time destinations. Based on our findings, we could conclude that when parents perceive that it is important for their child to participate regularly in physical activity and have positive attitudes (more benefits, less barriers) towards their children’s physical activity at primary school, children will be more likely to use active transport to leisure time destinations at secondary school level.

The findings of the present study could give some possible directions for future interventions promoting children’s active transport during the transition from primary to secondary school. Since an increase in home-school distance between primary and secondary school was found to be the key factor for children to switch to or keep using passive transport to school, future interventions should take that factor into account. To prevent that children switch to or maintain using passive transport, it is necessary to promote different possibilities at primary school for children and parents to use active transport when going to secondary school even if the home-school distance will increase. First, children who will be living at feasible distances from secondary school should be stimulated to walk or cycle to school. For children who will be living at non-feasible distances from secondary school, alternative possibilities should be provided by stimulating them to walk or cycle a part of their home-school trip instead of the entire trip (e.g. walking/cycling the first and/or last part of their home-school trip when they will take the public transport or by implementing drop-off spots located at a feasible walking distance from school when they are driven to school [38]). Furthermore, at primary school level, one should focus on providing safe neighborhoods, combined with programs for parents stimulating a positive social norm and positive attitudes towards physical activity because this can be effective to prevent that children will switch to or maintain using passive transport to leisure time destinations during the transition to secondary school. However, more large-scale longitudinal research regarding correlates of children’s transport during the transition from primary to secondary school is needed to confirm and elaborate the results of the present study.

The present study has some important strengths. To our knowledge, this is one of the first studies investigating the combination of individual, psychosocial and physical neighborhood environmental factors of children’s transport both to school and to leisure time destinations using a longitudinal design. Additionally, this is one of the first studies including measures of passive transport to determine different types regarding children’s switch to or maintenance of active/passive transport during the transition from primary to secondary school. Third, the present study also included factors at baseline (primary school level). This is of great value to inform future interventions, since it is important to target young children when developing appropriate interventions to prevent that children use passive transport at secondary school. Another strength of this study was the sampling across different suburban and urban schools in Flanders.

Some limitations of this study should be considered. First, this study has been conducted in a Belgian sample. Since active transport rates among Belgian children and adolescents are found to be higher compared to many other countries and continents [39], the results of this study cannot be generalized to other countries and continents. Children of low SES were also underrepresented in the present study. Another limitation involved the use of questionnaires which can induce social desirability. In this study, combined transport (i.e. public transport including walking to a bus stop) was classified as passive transport which could have induced bias in children’s active transport measures. We therefore recommend that future research should better disentangle both components (public transport versus walking to a bus stop) for instance by using detailed diaries or GPS. Fourth, a relative small sample size (n = 313) was used to conduct multinomial regression analyses. This resulted in limited power, did not make it possible to do further stratified analyses (e.g. subgroup analyses based on different home-school distances) and only relatively high effect sizes would result in significant findings in this sample. Therefore, we recommend that future longitudinal research, using similar multinomial logistic regression analyses, should aim to test a larger sample of participants. Fifth, only associations with physical neighborhood environmental factors were investigated in this study. Nevertheless, physical environmental factors outside children’s neighborhood might be important to determine choices of context-specific active and passive transport during the transition to secondary school. Further longitudinal research will probably need to focus on other contexts (e.g. environmental factors along routes) additional to the physical neighborhood environment. At last, the shortest distance between home and school was calculated and may possibly not represent the exact actual routes children followed. Future research could use GPS devices to track in detail the actual routes that children take and to objectively assess children’s transport to school or to leisure time destinations.

## Conclusions

A few individual, parental perceived psychosocial and physical neighborhood environmental factors were found to be significantly associated with children’s switch to or maintenance of active and passive transport during the transition from primary to secondary school. For transport to leisure time destinations, more and different associations were identified than for transport to school. The increase of the home-school distance was the key factor determining children’s switch to or maintenance of passive transport to school. For transport to leisure time destinations, parental perceived neighborhood safety from traffic and crime at primary school was an important factor for children to maintain using active transport to leisure time destinations. In addition to neighborhood safety, a positive social norm, positive attitudes (i.e. more benefits (e.g. improvement of health) and less barriers (e.g. lack of time)) towards their children’s physical activity at baseline were also important for children’s maintenance of active transport to leisure time destinations. The findings of the present study add to the limited evidence from longitudinal studies and can give some possible directions for future interventions promoting children’s active transport during the transition from primary to secondary school. To prevent that children will switch to or maintain using passive transport, it is necessary to promote different possibilities at primary school in order to deal with the increase in home-school distance during the transition to secondary school. Furthermore, providing safe neighborhoods, combined with programs for parents stimulating a positive social norm and positive attitudes towards physical activity at primary school level, can be effective to prevent that children switch to or maintain using passive transport during the transition to secondary school.

## Supporting Information

S1 File Child questionnaire. (DOCX)

S2 File Parental questionnaire. (DOCX)

S3 File SPSS Data-set. (SAV)
