# Supplementary material for: Which Socio-Ecological Factors Associate with a Switch to or Maintenance of Active and Passive Transport during the Transition from Primary to Secondary School?
Source: PLoS One. 2016 May 27;11(5):e0156531. doi: 10.1371/journal.pone.0156531 (PMC4883766; doi:10.1371/journal.pone.0156531)
Supplement: S1 File — (DOCX) [file pone.0156531.s001.docx]

**Supporting Information file 1.** **Child questionnaire**

**Socio-demographic information**

1. What is your birth date?
2. Are you a boy or a girl?

- Boy
- Girl

**Some questions about your physical activity**

By answering the following questions, face a ‘usual’ week (7 days)

1. How do you usually go to school?

- on foot
- by bike
- by car, by bus, train or tram

1. Do you usually walk to destinations on weekdays?

(e.g. to the sports club, to the bakery, to shops,..)

=> Do not include active transportation to school and walking as a sport.

O yes O no

1. Do you usually walk to destinations on weekend days?

(e.g. to the sports club, to the bakery, to shops,..)

=> Do not include walking as a sport.

O yes O no

1. Do you usually cycle to destinations on weekdays?

(e.g. to the sports club, to the bakery, to shops,..)

=>Do not include active transportation to school and cycling as a sport.

O yes O no

1. Do you usually cycle to destinations on weekend days?

(e.g. to the sports club, to the bakery, to shops,..)

=>Do not include cycling as a sport.

O yes O no
