# Supplementary material for: Which Socio-Ecological Factors Associate with a Switch to or Maintenance of Active and Passive Transport during the Transition from Primary to Secondary School?
Source: PLoS One. 2016 May 27;11(5):e0156531. doi: 10.1371/journal.pone.0156531 (PMC4883766; doi:10.1371/journal.pone.0156531)
Supplement: S2 File — (DOCX) [file pone.0156531.s002.docx]

**Supporting Information file 2.** **Parental questionnaire**

**Socio-demographic information**

1. What is the home address where your child mostly lives?

Street:

Number:

Bus:

ZIP code:

City:

1. What is the highest education level/diploma that the mother has obtained?

- Elementary education
- Vocational secondary education
- Technical secondary education
- General secondary education
- College education
- University education

1. What is the highest education level/diploma that the father has obtained?

- Elementary education
- Vocational secondary education
- Technical secondary education
- General secondary education
- College education
- University education

**Some questions about you and your child**

1. How frequently do you encourage your child to be physically active?

- Never
- Seldom
- Sometimes
- Often
- Very often

Please circle the answer that best applies to you.

1. My child has to participate regularly in physical activity.

- Strongly disagree
- Somewhat disagree
- Neither agree or disagree
- Somewhat agree
- Strongly agree

1. I am sure my child will be physically active if…
2. he/she has to get up early.

- Strongly disagree
- Somewhat disagree
- Neither agree or disagree
- Somewhat agree
- Strongly agree

1. his/her friends want to do something else.

- Strongly disagree
- Somewhat disagree
- Neither agree or disagree
- Somewhat agree
- Strongly agree

1. he/she has a lot of work for school.

- Strongly disagree
- Somewhat disagree
- Neither agree or disagree
- Somewhat agree
- Strongly agree

1. it is exhausting and difficult.

- Strongly disagree
- Somewhat disagree
- Neither agree or disagree
- Somewhat agree
- Strongly agree

1. My child thinks that doing sports is good because…
2. he/she improves his/her condition and health.

- Strongly disagree
- Somewhat disagree
- Neither agree or disagree
- Somewhat agree
- Strongly agree

1. he/she gets in contact with (new) friends.

- Strongly disagree
- Somewhat disagree
- Neither agree or disagree
- Somewhat agree
- Strongly agree

1. he/she enjoys being physically active.

- Strongly disagree
- Somewhat disagree
- Neither agree or disagree
- Somewhat agree
- Strongly agree

1. he/she can show that he/she is better in sports than others.

- Strongly disagree
- Somewhat disagree
- Neither agree or disagree
- Somewhat agree
- Strongly agree

1. he/she does not get bored if he/she is physically active.

- Strongly disagree
- Somewhat disagree
- Neither agree or disagree
- Somewhat agree
- Strongly agree

1. he/she loses weight.

- Strongly disagree
- Somewhat disagree
- Neither agree or disagree
- Somewhat agree
- Strongly agree

1. My child is not able to engage in sports because…
2. of lack of time.

- Strongly disagree
- Somewhat disagree
- Neither agree or disagree
- Somewhat agree
- Strongly agree

1. he/she does not enjoy sports.

- Strongly disagree
- Somewhat disagree
- Neither agree or disagree
- Somewhat agree
- Strongly agree

1. he/she is not good in doing sports.

- Strongly disagree
- Somewhat disagree
- Neither agree or disagree
- Somewhat agree
- Strongly agree

1. my child does not have transportation to engage in sports.

- Strongly disagree
- Somewhat disagree
- Neither agree or disagree
- Somewhat agree
- Strongly agree

1. he/she is not allowed to sport by his/her parents.

- Strongly disagree
- Somewhat disagree
- Neither agree or disagree
- Somewhat agree
- Strongly agree

**Your neighborhood**

While thinking about the places where people live in your neighborhood, please circle an answer for each of the following questions. Your neighborhood is the local area around your home, within a 10-15 minute walk in any direction.

1. How common are different types of residences present in your neighborhood?
2. How common are separate or standalone one family homes in your neighborhood?

- None
- A few
- About half
- A lot
- All

1. How common are connected townhouses or row houses in your neighborhood?

- None
- A few
- About half
- A lot
- All

1. How common are apartments in your neighborhood?

- None
- A few
- About half
- A lot
- All

1. Please circle the time it would take your child to walk to each place, regardless of whether your child goes there. About how long does it take (for your child) to walk from your home to…
2. Grocery store

- > 30 min
- 21-30 min
- 11-20 min
- 6-10 min
- 1-5 min

1. Supermarket

- > 30 min
- 21-30 min
- 11-20 min
- 6-10 min
- 1-5 min

1. Bakery

- > 30 min
- 21-30 min
- 11-20 min
- 6-10 min
- 1-5 min

1. Butchery

- > 30 min
- 21-30 min
- 11-20 min
- 6-10 min
- 1-5 min

1. Convenience store

- > 30 min
- 21-30 min
- 11-20 min
- 6-10 min
- 1-5 min

1. Bank

- > 30 min
- 21-30 min
- 11-20 min
- 6-10 min
- 1-5 min

1. Library

- > 30 min
- 21-30 min
- 11-20 min
- 6-10 min
- 1-5 min

1. My school/ school of my child

- > 30 min
- 21-30 min
- 11-20 min
- 6-10 min
- 1-5 min

1. Please circle the answer that best applies to the neighborhood where you and your child live.
2. The streets in our neighborhood have many cul-de-sacs (dead end streets).

- Strongly disagree
- Somewhat disagree
- Neither agree or disagree
- Somewhat agree
- Strongly agree

1. There are a lot of crossroads in my neighborhood.

- Strongly disagree
- Somewhat disagree
- Neither agree or disagree
- Somewhat agree
- Strongly agree

1. Please circle the answer that best applies to the neighborhood where you and your child live. Both local and within walking distance mean within a 10-15 minute walk from your home.
2. In my neighborhood it’s easy (for my child) to walk to school.

- Strongly disagree
- Somewhat disagree
- Neither agree or disagree
- Somewhat agree
- Strongly agree

1. There are many places (for my child) to go (alone or with someone) within easy walking distance of my home.

- Strongly disagree
- Somewhat disagree
- Neither agree or disagree
- Somewhat agree
- Strongly agree

1. In my neighborhood it’s easy (for my child) to get from place to place (for example, freeways, railway lines, rivers).

- Strongly disagree
- Somewhat disagree
- Neither agree or disagree
- Somewhat agree
- Strongly agree

1. In my neighborhood it’s easy (for my child) to walk to a playground, park or skate park from my house.

- Strongly disagree
- Somewhat disagree
- Neither agree or disagree
- Somewhat agree
- Strongly agree

1. Please circle the answer that best applies to the neighborhood where you and your child live.
2. There are sidewalks on most of the streets in my neighborhood.

- Strongly disagree
- Somewhat disagree
- Neither agree or disagree
- Somewhat agree
- Strongly agree

1. There are cycle lanes on most of the streets in my neighborhood.

- Strongly disagree
- Somewhat disagree
- Neither agree or disagree
- Somewhat agree
- Strongly agree

1. Cycle lanes are separated from the road/traffic in my neighborhood by parked cars or grass.

- Strongly disagree
- Somewhat disagree
- Neither agree or disagree
- Somewhat agree
- Strongly agree

1. There are bicycle racks in my neighborhood (at shops, schools, transit stops, …).

- Strongly disagree
- Somewhat disagree
- Neither agree or disagree
- Somewhat agree
- Strongly agree

1. At night the sidewalks are well-lit in my neighborhood.

- Strongly disagree
- Somewhat disagree
- Neither agree or disagree
- Somewhat agree
- Strongly agree

1. The sidewalks are well maintained in my neighborhood.

- Strongly disagree
- Somewhat disagree
- Neither agree or disagree
- Somewhat agree
- Strongly agree

1. At night the cycle lanes are well-lit in my neighborhood.

- Strongly disagree
- Somewhat disagree
- Neither agree or disagree
- Somewhat agree
- Strongly agree

1. The cycle lanes are well maintained in my neighborhood.

- Strongly disagree
- Somewhat disagree
- Neither agree or disagree
- Somewhat agree
- Strongly agree

1. Playground and parks are well maintained in my neighborhood.

- Strongly disagree
- Somewhat disagree
- Neither agree or disagree
- Somewhat agree
- Strongly agree

1. Please circle the answer that best applies to the neighborhood where you and your child live.
2. There are trees along the streets in my neighborhood.

- Strongly disagree
- Somewhat disagree
- Neither agree or disagree
- Somewhat agree
- Strongly agree

1. There are many beautiful natural things (for my child) to look at in my neighborhood (e.g. gardens, views).

- Strongly disagree
- Somewhat disagree
- Neither agree or disagree
- Somewhat agree
- Strongly agree

1. There are many buildings/homes in our neighborhood that are nice (for my child) to look at.

- Strongly disagree
- Somewhat disagree
- Neither agree or disagree
- Somewhat agree
- Strongly agree

1. Please circle the answer that best applies to the neighborhood where you and your child live.
2. There is so much traffic along nearby streets that it makes it difficult or unpleasant (for my child) to walk (alone or with someone) in my neighborhood.

- Strongly disagree
- Somewhat disagree
- Neither agree or disagree
- Somewhat agree
- Strongly agree

1. There is so much traffic along nearby streets that it makes it difficult or unpleasant for my child to cycle (alone or with someone) in my neighborhood.

- Strongly disagree
- Somewhat disagree
- Neither agree or disagree
- Somewhat agree
- Strongly agree

1. The speed of traffic on most nearby streets is usually slow.

- Strongly disagree
- Somewhat disagree
- Neither agree or disagree
- Somewhat agree
- Strongly agree

1. Our neighborhood streets have good lighting at night.

- Strongly disagree
- Somewhat disagree
- Neither agree or disagree
- Somewhat agree
- Strongly agree

1. There are crosswalks and signals to help walkers cross busy streets in our neighborhood.

- Strongly disagree
- Somewhat disagree
- Neither agree or disagree
- Somewhat agree
- Strongly agree

1. It’s safe for my child to play on the street in my neighborhood.

- Strongly disagree
- Somewhat disagree
- Neither agree or disagree
- Somewhat agree
- Strongly agree

1. There is a low crime rate in our neighborhood.

- Strongly disagree
- Somewhat disagree
- Neither agree or disagree
- Somewhat agree
- Strongly agree

1. I am worried about (letting my child) play(ing) outside alone around my home (e.g. yard, driveway, apartment common area) because I am afraid of them being taken or hurt by a stranger.

- Strongly disagree
- Somewhat disagree
- Neither agree or disagree
- Somewhat agree
- Strongly agree

1. I am worried about (letting my child) be(ing) alone in a local or nearby park because I am afraid of them being taken or hurt by a stranger.

- Strongly disagree
- Somewhat disagree
- Neither agree or disagree
- Somewhat agree
- Strongly agree

1. My bike is securely locked in my neighborhood.

- Strongly disagree
- Somewhat disagree
- Neither agree or disagree
- Somewhat agree
- Strongly agree

1. Please circle the time it would take your child to cycle to each place, regardless of whether your child goes there. How long does it take (for your child) to cycle from your home to…
2. Indoor recreation facility

- > 30 min
- 21-30 min
- 11-20 min
- 6-10 min
- 1-5 min

1. Outdoor recreation facility

- > 30 min
- 21-30 min
- 11-20 min
- 6-10 min
- 1-5 min

1. Public park

- > 30 min
- 21-30 min
- 11-20 min
- 6-10 min
- 1-5 min

1. Swimming pool

- > 30 min
- 21-30 min
- 11-20 min
- 6-10 min
- 1-5 min

1. Public playground

- > 30 min
- 21-30 min
- 11-20 min
- 6-10 min
- 1-5 min
